# Post–COVID-19 Symptoms and Conditions Among Children and Adolescents — United States, March 1, 2020–January 31, 2022

**DOI:** 10.15585/mmwr.mm7131a3

**Published:** 2022-08-05

**Authors:** Lyudmyla Kompaniyets, Lara Bull-Otterson, Tegan K. Boehmer, Sarah Baca, Pablo Alvarez, Kai Hong, Joy Hsu, Aaron M. Harris, Adi V. Gundlapalli, Sharon Saydah

**Affiliations:** ^1^CDC COVID-19 Emergency Response Team; ^2^GAP Solutions Inc, Herndon, Virginia.

Post–COVID-19 (post-COVID) symptoms and conditions* are new, recurring, or ongoing health problems that occur 4 or more weeks after infection with SARS-CoV-2 (the virus that causes COVID-19). Previous studies have characterized and estimated the incidence of post-COVID conditions among adults (*1*,*2*), but data among children and adolescents are limited (*3**–**8*). Using a large medical claims database, CDC assessed nine potential post-COVID signs and symptoms (symptoms) and 15 potential post-COVID conditions among 781,419 U.S. children and adolescents aged 0–17 years with laboratory-confirmed COVID-19 (patients with COVID-19) compared with 2,344,257 U.S. children and adolescents without recognized COVID-19 (patients without COVID-19) during March 1, 2020–January 31, 2022. The analysis identified several symptoms and conditions with elevated adjusted hazard ratios among patients with COVID-19 (compared with those without). The highest hazard ratios were recorded for acute pulmonary embolism (adjusted hazard ratio [aHR] = 2.01), myocarditis and cardiomyopathy (1.99), venous thromboembolic event (1.87), acute and unspecified renal failure (1.32), and type 1 diabetes (1.23), all of which were rare or uncommon in this study population. Conversely, symptoms and conditions that were most common in this study population had lower aHRs (near or below 1.0). Patients with COVID-19 were less likely than were patients without to experience respiratory signs and symptoms, symptoms of mental conditions, muscle disorders, neurological conditions, anxiety and fear-related disorders, mood disorders, and sleeping disorders. COVID-19 prevention strategies, including vaccination for all eligible children and adolescents, are critical to prevent SARS-CoV-2 infection and subsequent illness, including post-COVID symptoms and conditions (*9*).

CDC analyzed linked medical claims and commercial laboratory data for persons with a health care encounter possibly related to COVID-19.^†^ Analyses were restricted to children and adolescents aged 0–17 years who were continuously enrolled in a health insurance plan during March 1, 2019–January 31, 2022. Children and adolescents aged 0–17 years with laboratory-confirmed COVID-19 and those without recognized COVID-19^§^ were matched 1:3 based on age at encounter, sex, and month of index date.^¶^ Patients were followed for a minimum of 60 days and a maximum of 365 days or until January 31, 2022, whichever occurred first. Scientific literature on symptoms and conditions associated with post-COVID illness among children or adults was reviewed (*1*–*5*). Symptoms and conditions were identified by the first occurrence and classified based on the *International Classification of Diseases, Tenth Revision, Clinical Modification* (ICD-10-CM) codes documented 31–365 days after the index date but not during the 7–365 days preceding the index date.**

The incidences (occurrence per 100,000 person-years) of nine potential post-COVID symptoms and 15 potential post-COVID conditions among children and adolescents aged 0–17 years were calculated. Separate Cox proportional hazards models were used to estimate aHRs for each symptom and condition, after excluding persons with that particular symptom or condition during the 7–365 days preceding the index date.^††^ All models were adjusted for age, sex, race, U.S. Census Bureau region, payor type, previous medical complexity (*10*), and previous hospitalization.^§§^ The same models were estimated separately for three age groups (2–4, 5–11, and 12–17 years).^¶¶^ A sensitivity analysis was performed to assess the incidences of potential post-COVID symptoms and conditions among children and adolescents aged 0–17 years who had not experienced any of the 24 assessed symptoms or conditions before the index date.*** Finally, incidence of each symptom and condition among patients with COVID-19 was plotted against aHRs from the main analysis. Analyses were conducted using R software (version 4.1.0; R Foundation); p-values <0.05 were considered statistically significant. This activity was reviewed by CDC and conducted consistent with applicable federal law and CDC policy.^†††^

During March 1, 2020–January 31, 2022, a total of 781,419 patients aged 0–17 years with COVID-19 and 2,344,257 patients aged 0–17 years without COVID-19 were identified (Table 1). The median age of both patients with and without COVID-19 was 12 years, and 50.0% in both groups were female; 72.2% of patients with COVID-19 were enrolled in Medicaid managed care, compared with 70.6% of patients without COVID-19. Patients without COVID-19 had a higher prevalence of previous hospitalization (4.5%) and complex chronic disease (15.6%), than did patients with COVID-19 (3.6% and 11.7%, respectively).

**TABLE 1:** Characteristics of children and adolescents aged 0–17 years with and without COVID-19 — HealthVerity medical claims database, United States, March 1, 2020–January 31, 2022

Characteristic*	No. (%)			
	All patients^†^		Patients without previous symptoms or conditions^†^	
	Patients without COVID-19^§^	Patients with COVID-19^§^	Patients without COVID-19^§^	Patients with COVID-19^§^
**Total**	**2,344,257**	**781,419**	**792,672**	**396,336**
**Sex**				
Female	1,172,481 (50.0)	390,827 (50.0)	394,536 (49.8)	197,268 (49.8)
Male	1,171,776 (50.0)	390,592 (50.0)	398,136 (50.2)	199,068 (50.2)
**Age group, yrs**				
Median (IQR)	12 (8–15)	12 (8–15)	12 (8–15)	12 (8–15)
<2	2,952 (0.1)	984 (0.1)	564 (0.1)	282 (0.1)
2–4	155,190 (6.6)	51,730 (6.6)	48,550 (6.1)	24,275 (6.1)
5–11	904,284 (38.6)	301,428 (38.6)	316,010 (39.9)	158,005 (39.9)
12–17	1,281,831 (54.7)	427,277 (54.7)	427,548 (53.9)	213,774 (53.9)
**Race^¶^**				
Asian	74,943 (3.2)	23,241 (3.0)	26,039 (3.3)	12,213 (3.1)
Black or African American	566,891 (24.2)	196,887 (25.2)	198,705 (25.1)	101,839 (25.7)
White	1,178,288 (50.3)	382,371 (48.9)	381,841 (48.2)	189,895 (47.9)
Other	73,401 (3.1)	24,648 (3.2)	26,044 (3.3)	12,889 (3.3)
Unknown or unavailable	450,734 (19.2)	154,272 (19.7)	160,043 (20.2)	79,500 (20.1)
**Payor type**				
Commercial	666,068 (28.4)	214,371 (27.4)	244,047 (30.7)	118,300 (29.8)
Medicaid	1,655,886 (70.6)	563,860 (72.2)	541,415 (68.3)	276,422 (69.7)
Medicare Advantage	13,466 (0.6)	1,277 (0.2)	4,415 (0.6)	676 (0.2)
Unknown or unavailable	8,837 (0.4)	1,911 (0.2)	2,795 (0.4)	938 (0.2)
U.S. Census Bureau region**				
Northeast	283,916 (12.1)	86,436 (11.1)	79,015 (10.0)	44,770 (11.3)
Midwest	527,527 (22.5)	132,879 (17.0)	167,233 (21.1)	68,471 (17.3)
South	1,160,472 (49.5)	448,844 (57.4)	397,624 (50.2)	217,886 (55.0)
West	372,342 (15.9)	113,260 (14.5)	148,800 (18.8)	65,209 (16.5)
**Hospitalization during 7–365 days preceding index date**				
Yes	104,768 (4.5)	28,294 (3.6)	8,030 (1.0)	4,007 (1.0)
No	2,239,489 (95.5)	753,125 (96.4)	784,642 (99.0)	392,329 (99.0)
**Medical complexity during 7–365 days preceding index date**				
No chronic disease	1,328,582 (56.7)	506,026 (64.8)	672,355 (84.8)	333,882 (84.2)
Non-complex chronic disease	649,710 (27.7)	184,188 (23.6)	95,337 (12.0)	50,980 (12.9)
Complex chronic disease	365,965 (15.6)	91,205 (11.7)	24,980 (3.2)	11,474 (2.9)

**Abbreviation**: ICD-10-CM = *International Classification of Diseases, Tenth Revision, Clinical Modification.*

* Categories might not sum to 100% because of rounding or missing values.

^†^ Columns 2 and 3 describe the main cohort: patients with COVID-19 and patients without COVID-19 who were matched 1:3 based on age, sex, and month of the index date (for patients with COVID-19, the date of either the first claim with a COVID-19 diagnosis code or the first positive SARS-CoV-2 test result, whichever occurred first; for patients without COVID-19, the date of a randomly selected claim during the month in which the patient without COVID-19 was matched to a patient with COVID-19). Columns 4 and 5 describe a cohort of patients with none of the 24 assessed symptoms or conditions during 7–365 days before the index date; patients with COVID-19 and patients without COVID-19 were matched 1:2 based on age, sex, and month of the index date (for patients with COVID-19, the date of either the first claim with a COVID-19 diagnosis code or the first positive SARS-CoV-2 test result, whichever occurred first; for patients without COVID-19, the date of the first negative SARS-CoV-2 test result, first health care encounter possibly related to COVID-19, or the first claim during the pandemic period if other dates were not available).

^§^ The cohort consisted of children and adolescents aged 0–17 years with continuous enrollment in an insurance plan during March 1, 2019–January 31, 2022, identified within a subset of CDC-licensed HealthVerity data that included persons with a health care encounter possibly related to COVID-19. Patients with COVID-19 were selected from patients who received a positive SARS-CoV-2 test result during March 2020–November 2021 or an ICD-10-CM code of B97.29 during March–April 2020 or U07.1 code during April 2020–November 2021. Patients without COVID-19 were selected after excluding patients who had an ICD-10-CM code related to COVID-19 (A41.89, B34.2, B97.21, B97.29, B94.8, J12.81, J12.82, J12.89, M30.3, M35.81, U07.1, or U07.2), a positive SARS-CoV-2 test result, or received treatment for COVID-19 (casirivimab/imdevimab, etesevimab/bamlanivimab, sotrovimab, bebtelovimab, nirmatrelvir, molnupiravir, or remdesivir) at any point during the study period. Vaccination status of patients was not included for this analysis.

**^¶^** Analysis did not include ethnicity.

** U.S. Census Bureau regions:* Northeast*: Connecticut, Maine, Massachusetts, New Hampshire, New Jersey, New York, Pennsylvania, Rhode Island, and Vermont; *Midwest*: Illinois, Indiana, Iowa, Kansas, Michigan, Minnesota, Missouri, Nebraska, North Dakota, Ohio, South Dakota, and Wisconsin; *South*: Alabama, Arkansas, Delaware, District of Columbia, Florida, Georgia, Kentucky, Louisiana, Maryland, Mississippi, North Carolina, Oklahoma, South Carolina, Tennessee, Texas, Virginia, and West Virginia; *West*: Alaska, Arizona, California, Colorado, Hawaii, Idaho, Montana, Nevada, New Mexico, Oregon, Utah, Washington, and Wyoming.

Patients with COVID-19 were significantly more likely than were those without to develop the following assessed post-COVID symptoms: smell and taste disturbances (aHR = 1.17), circulatory signs and symptoms (1.07), malaise and fatigue (1.05), and musculoskeletal pain (1.02) (Table 2). Patients with COVID-19 were also more likely than were those without to develop the following assessed post-COVID conditions: acute pulmonary embolism (2.01), myocarditis and cardiomyopathy (1.99), venous thromboembolic event (1.87), acute and unspecified renal failure (1.32), type 1 diabetes (1.23), coagulation and hemorrhagic disorders (1.18), type 2 diabetes (1.17), and cardiac dysrhythmias (1.16). Patients with COVID-19 were less likely than were those without to experience respiratory signs and symptoms (0.91), symptoms of mental conditions (0.91), sleeping disorders (0.91), neurological conditions (0.94), anxiety and fear-related disorders (0.85), mood disorders (0.78), and muscle disorders (0.94); no significant associations were found for the remaining five symptoms and conditions.

**TABLE 2:** Incidence* and adjusted hazard ratios of selected potential post–COVID-19 symptoms and conditions among children and adolescents aged 0–17 years with and without COVID-19 — HealthVerity medical claims database, United States, March 1, 2020–January 31, 2022

Outcome	All patients^†^			Patients without previous symptoms or conditions^†^		
	No. (incidence)*		aHR (95% CI)^§^	No. (incidence)*		aHR (95% CI)^§^
	Patients without COVID-19	Patients with COVID-19		Patients without COVID-19	Patients with COVID-19	
**Symptom**						
Smell and taste disturbances	5,028 (296)	1,924 (340)	1.17 (1.11–1.24)^¶^	1,173 (205)	715 (250)	1.21 (1.11–1.33)^¶^
Circulatory signs and symptoms	80,900 (5,092)	27,207 (5,126)	1.07 (1.05–1.08)^¶^	18,729 (3,334)	10,518 (3,727)	1.12 (1.09–1.14)^¶^
Malaise and fatigue	74,908 (4,659)	24,970 (4,648)	1.05 (1.03–1.06)^¶^	15,712 (2,784)	8,964 (3,168)	1.13 (1.10–1.16)^¶^
Musculoskeletal pain	201,899 (14,819)	67,744 (14,800)	1.02 (1.02–1.03)^¶^	62,417 (11,647)	34,460 (12,662)	1.09 (1.07–1.10)^¶^
Dizziness and syncope	48,976 (2,993)	15,731 (2,876)	1.01 (0.99–1.03)	10,890 (1,923)	5,630 (1,980)	1.03 (0.99–1.06)
Gastrointestinal and esophageal disorders	94,395 (6,195)	30,266 (5,898)	1.01 (0.99–1.02)	22,411 (4,021)	11,686 (4,151)	1.03 (1.00–1.05)
Sleeping disorders	51,227 (3,203)	14,011 (2,588)	0.91 (0.90–0.93)^¶^	9,138 (1,616)	4,238 (1,488)	0.92 (0.89–0.95)^¶^
Respiratory signs and symptoms	283,139 (23,456)	85,279 (20,948)	0.91 (0.91–0.92)^¶^	80,364 (15,200)	47,690 (17,796)	1.16 (1.14–1.17)^¶^
Symptoms of mental conditions	47,138 (2,906)	12,944 (2,364)	0.91 (0.89–0.93)^¶^	9,268 (1,637)	4,529 (1,591)	0.97 (0.94–1.00)
**Condition**						
Acute pulmonary embolism	224 (13)	131 (23)	2.01 (1.62–2.50)^¶^	43 (8)	36 (13)	1.74 (1.12–2.72)^¶^
Myocarditis and cardiomyopathy	1,172 (69)	692 (122)	1.99 (1.81–2.19)^¶^	224 (39)	264 (92)	2.34 (1.96–2.79)^¶^
Venous thromboembolic event	315 (18)	164 (29)	1.87 (1.54–2.26)^¶^	51 (9)	37 (13)	1.48 (0.97–2.26)
Acute and unspecified renal failure	2,116 (124)	788 (139)	1.32 (1.22–1.43)^¶^	347 (61)	223 (78)	1.30 (1.10–1.54)^¶^
Type 1 diabetes	2,080 (123)	792 (140)	1.23 (1.13–1.33)^¶^	641 (112)	349 (122)	1.10 (0.96–1.25)
Coagulation and hemorrhagic disorders	4,454 (263)	1,582 (280)	1.18 (1.12–1.25)^¶^	849 (148)	537 (188)	1.26 (1.14–1.41)^¶^
Type 2 diabetes	6,197 (366)	2,170 (384)	1.17 (1.11–1.23)^¶^	1,210 (212)	729 (255)	1.19 (1.09–1.31)^¶^
Cardiac dysrhythmias	13,031 (774)	4,595 (817)	1.16 (1.12–1.20)^¶^	2,391 (419)	1,442 (504)	1.20 (1.13–1.28)^¶^
Cerebrovascular disease	441 (26)	149 (26)	1.20 (1.00–1.45)	67 (12)	28 (10)	0.84 (0.54–1.30)
Chronic kidney disease	1,105 (65)	321 (57)	1.07 (0.95–1.22)	171 (30)	81 (28)	0.99 (0.76–1.29)
Asthma	82,105 (5,625)	27,327 (5,557)	1.00 (0.99–1.01)	26,470 (4,785)	12,751 (4,533)	0.93 (0.91–0.95)^¶^
Muscle disorders	23,655 (1,424)	6,807 (1,222)	0.94 (0.91–0.96)^¶^	4,075 (715)	2,109 (738)	1.03 (0.98–1.09)
Neurological conditions	64,436 (4,077)	18,681 (3,485)	0.94 (0.92–0.95)^¶^	12,954 (2,298)	6,513 (2,295)	0.99 (0.96–1.02)
Anxiety and fear-related disorders	112,234 (7,686)	31,274 (6,107)	0.85 (0.84–0.86)^¶^	28,624 (5,166)	13,016 (4,634)	0.90 (0.88–0.91)^¶^
Mood disorders	23,108 (1,406)	5,248 (944)	0.78 (0.75–0.80)^¶^	3,656 (642)	1,531 (535)	0.83 (0.78–0.88)^¶^

**Abbreviation**: aHR = adjusted hazard ratio.

* Occurrences per 100,000 person-years.

^†^ Columns 2, 3, and 4 represent analyses of incidences and aHRs obtained after 1:3 matching of patients with COVID-19 and patients without COVID-19. Incidences and aHRs for each symptom or condition were calculated after excluding patients who had that particular symptom or condition during 7–365 days before the index date (for patients with COVID-19, the date of either the first claim with a COVID-19 diagnosis code or the first positive SARS-CoV-2 test result, whichever occurred first; for patients without COVID-19, the date of a randomly selected claim during the month in which the patient without COVID-19 was matched to a patient with COVID-19). Columns 5, 6, and 7 represent incidences and aHRs obtained after 1:2 matching of patients with COVID-19 and those without who had not experienced any of the 24 assessed symptoms or conditions during 7–365 days before the index date (for patients with COVID-19, the date of either the first claim with a COVID-19 diagnosis code or the first positive SARS-CoV-2 test result, whichever occurred first; for patients without COVID-19, the date of the first negative SARS-CoV-2 test result, first health care encounter possibly related to COVID-19, or the first claim during the pandemic period if other dates were not available).

^§^ Each aHR was obtained from a single Cox proportional hazards model, with the specific symptom or condition as the outcome and the following covariates: presence of COVID-19, age group, sex, race, U.S. Census Bureau region, payor type, previous medical complexity, and previous hospitalization.

^¶^ P-value <0.05.

In age-stratified analysis of three age groups (2–4, 5–11, and 12–17 years), the unadjusted incidences of symptoms and conditions differed by age group (Supplementary Table, https://stacks.cdc.gov/view/cdc/118760). Among children aged 2–4 years, the highest aHRs for patients with COVID-19 compared with patients without COVID-19 were for myocarditis and cardiomyopathy (aHR  = 2.39), acute and unspecified renal failure (1.52), and coagulation and hemorrhagic disorders (1.47) (Table 3). Unlike other age groups, children aged 2–4 years had higher rates of asthma diagnosis (1.12) and respiratory signs and symptoms (1.07) after COVID-19. Among children aged 5–11 years, the highest aHRs for patients with COVID-19 compared with those without were for myocarditis and cardiomyopathy (2.84), venous thromboembolic event (2.69), and acute and unspecified renal failure (1.38). Among patients aged 12–17 years, the highest aHRs for those with COVID-19 compared with those without were for acute pulmonary embolism (2.03), myocarditis and cardiomyopathy (1.66), and venous thromboembolic event (1.52).

**TABLE 3:** Adjusted hazard ratios of selected potential post–COVID-19 symptoms and conditions among children and adolescents aged 2–17 years with and without COVID-19, by age group — HealthVerity medical claims database, United States, March 1, 2020–January 31, 2022

Outcome	Adjusted hazard ratio (95% CI)*		
	Aged 2–4 yrs	Aged 5–11 yrs	Aged 12–17 yrs
**Symptom**			
Smell and taste disturbances	1.22 (0.70–2.15)	0.94 (0.83–1.07)	1.23 (1.16–1.31)^†^
Circulatory signs and symptoms	1.17 (1.12–1.23)^†^	1.11 (1.08–1.13)^†^	1.04 (1.02–1.06)^†^
Malaise and fatigue	1.13 (1.05–1.22)^†^	1.08 (1.05–1.12)^†^	1.03 (1.01–1.04)^†^
Musculoskeletal pain	1.16 (1.10–1.21)^†^	1.06 (1.04–1.07)^†^	1.00 (0.99–1.01)
Dizziness and syncope	1.08 (0.90–1.29)	1.03 (0.99–1.08)	1.00 (0.98–1.02)
Gastrointestinal and esophageal disorders	1.15 (1.10–1.20)^†^	1.02 (1.00–1.04)^†^	0.97 (0.95–0.99)^†^
Sleeping disorders	0.99 (0.93–1.06)	0.89 (0.86–0.92)^†^	0.91 (0.89–0.94)^†^
Respiratory signs and symptoms	1.07 (1.04–1.10)^†^	0.93 (0.92–0.94)^†^	0.88 (0.87–0.89)^†^
Symptoms of mental conditions	1.03 (0.97–1.10)	0.92 (0.90–0.95)^†^	0.89 (0.86–0.91)^†^
**Condition**			
Acute pulmonary embolism	—^§^	—^§^	2.03 (1.61–2.56)^†^
Myocarditis and cardiomyopathy	2.39 (1.57–3.65)^†^	2.84 (2.39–3.37)^†^	1.66 (1.48–1.88)^†^
Venous thromboembolic event	—^§^	2.69 (1.73–4.19)^†^	1.52 (1.22–1.91)^†^
Acute and unspecified renal failure	1.52 (1.07–2.14)^†^	1.38 (1.16–1.63)^†^	1.27 (1.15–1.40)^†^
Type 1 diabetes	1.01 (0.57–1.78)	1.31 (1.13–1.53)^†^	1.20 (1.09–1.33)^†^
Coagulation and hemorrhagic disorders	1.47 (1.20–1.80)^†^	1.28 (1.15–1.43)^†^	1.10 (1.03–1.19)^†^
Type 2 diabetes	1.24 (0.85–1.81)	1.14 (1.02–1.28)^†^	1.18 (1.11–1.24)^†^
Cardiac dysrhythmias	1.44 (1.22–1.70)^†^	1.23 (1.14–1.32)^†^	1.12 (1.08–1.17)^†^
Cerebrovascular disease	1.66 (0.85–3.23)	1.14 (0.79–1.64)	1.18 (0.93–1.48)
Chronic kidney disease	0.86 (0.54–1.36)	1.04 (0.83–1.31)	1.12 (0.96–1.31)
Asthma	1.12 (1.07–1.18)^†^	1.02 (1.00–1.05)^†^	0.96 (0.94–0.98)^†^
Muscle disorders	0.87 (0.77–0.98)^†^	0.86 (0.82–0.91)^†^	0.96 (0.93–0.99)^†^
Neurological conditions	0.98 (0.93–1.04)	0.96 (0.93–0.98)^†^	0.91 (0.89–0.93)^†^
Anxiety and fear-related disorders	0.91 (0.83–1.00)	0.86 (0.83–0.88)^†^	0.84 (0.82–0.85)^†^
Mood disorders	0.82 (0.62–1.08)	0.73 (0.69–0.77)^†^	0.80 (0.77–0.83)^†^

* Each adjusted hazard ratio was obtained from a single Cox proportional hazards model stratified by age group, with the specific symptom or condition as the outcome and the following covariates: presence of COVID-19, age (continuous variable), sex, race, U.S. Census Bureau region, payor type, previous medical complexity, and previous hospitalization.

^†^ P-value <0.05

^§^ Age-stratified analyses were only performed when there were at least 10 patients with COVID-19 and at least 10 patients without COVID-19 in that age group with the specific symptom or condition.

The sensitivity analysis of 396,336 patients with COVID-19 and 792,672 matched patients without COVID-19 (without previous symptoms or conditions of interest) found that patients in both groups were healthier at baseline than their counterparts in the main cohort; 84.2% of persons with COVID-19 and 84.8% patients without COVID-19 had no previous documentation of chronic disease, compared with 64.8% and 56.7%, respectively in the main cohort (Table 1). Higher rates of five symptoms and six conditions among patients with COVID-19 compared with those without were found in the sensitivity analysis, whereas the main analysis found higher rates of four symptoms and eight conditions. In the sensitivity analysis, aHRs for type 1 diabetes and venous thromboembolic event were not statistically significant, and the aHR for respiratory signs and symptoms was elevated (1.16) (Table 2).

Analysis of the relationship between incidence rates among patients with COVID-19 and aHRs found that five post-COVID conditions with the highest aHRs had low incidence rates, ranging from 23 (acute pulmonary embolism) to 140 (type 1 diabetes) per 100,000 person-years (Supplementary Figure, https://stacks.cdc.gov/view/cdc/118761). Conversely, this analysis found that five symptoms and conditions with the highest incidence rates among patients with COVID-19 had lower aHRs (near or below 1.0): respiratory signs and symptoms (0.91), musculoskeletal pain (1.02), anxiety and fear-related disorders (0.85), gastrointestinal and esophageal disorders (1.01), and asthma (1.00).

## Discussion

This analysis found increased incidence rates of several symptoms and conditions during the 31–365 days after a diagnosis of COVID-19 among children and adolescents aged 0–17 years. The highest aHRs were associated with potentially serious conditions, such as acute pulmonary embolism, myocarditis and cardiomyopathy, venous thromboembolic event, acute and unspecified renal failure, and type 1 diabetes. These conditions with the highest aHRs were rare or uncommon among children and adolescents in this analysis. Some of the study’s findings are consistent with previous evidence of elevated risk for new onset of diabetes (*5*), myocarditis (*6*), and certain symptoms (*4*), whereas other conditions (acute pulmonary embolism, venous thromboembolic event, acute renal failure, coagulation and hemorrhagic disorders, and cardiac dysrhythmias) have not been previously reported as post-COVID conditions among children and adolescents.

Several symptoms and conditions (respiratory signs and symptoms, mental health symptoms and conditions, neurological conditions, muscle disorders, and sleeping disorders) were less likely to occur among patients with COVID-19 than among patients without COVID-19. Reasons for these observed associations are likely multifactorial, and might be, in part, because patients without COVID-19 were selected from a cohort of patients with a health care encounter possibly related to COVID-19 and were less healthy than were patients with COVID-19 at baseline. Although most of the symptoms and conditions selected for the analysis were based on those observed in previous post-COVID studies, they are not unique to patients with a history of COVID-19, and many are common among children and adolescents. A United Kingdom study found a high prevalence of poor mental health and wellbeing among all children and adolescents aged 11–17 years during the pandemic, but no difference among those with positive and negative SARS-CoV-2 test results (*7*). Respiratory signs and symptoms were less likely to occur among patients with COVID-19 than among those without in the main cohort. The opposite result was found in a subset of children aged 2–4 years and in a cohort of children and adolescents with no previous symptoms or conditions of interest; new respiratory signs and symptoms were more likely to occur among children and adolescents who had COVID-19, compared with those without a history of COVID-19.

The findings in this report are subject to at least seven limitations. First, the definitions of potential post-COVID symptoms and conditions are subject to misclassification bias because the symptoms and conditions were defined by a single ICD-10-CM code and no information on laboratory assessments or degree of severity was available. Second, because the incidence date of a symptom or a condition was based on the first occurrence of the ICD-10-CM code, the actual incidence date of that symptom or condition might have occurred prior to COVID-19. Third, patients infected with SARS-CoV-2 without a documented COVID-19 diagnosis or positive test result might have been misclassified as not having had COVID-19, potentially reducing the magnitude of observed associations. Fourth, the aHR estimates might be reduced because patients without COVID-19 were patients with a health care encounter possibly related to COVID-19. Fifth, because patients’ vaccination status was likely underreported in this dataset, this analysis was not adjusted for previous receipt of COVID-19 vaccines. Sixth, although this study relied on statistical significance for interpreting the increased rates of symptoms and conditions, further understanding of the clinical significance of the observed associations, including whether these symptoms and conditions are transient or chronic, is necessary. Finally, generalizability might be limited because the analysis was restricted to children and adolescents aged 0–17 years included in a medical claims database, approximately 70% of whom were enrolled in Medicaid managed care; therefore, findings are not necessarily representative of all children and adolescents with COVID-19 or of those who do not seek health care.

These findings can be used to apprise health care professionals and caregivers about new symptoms and conditions that occur among children and adolescents in the months after SARS-CoV-2 infection. COVID-19 prevention strategies, including vaccination for all eligible persons aged ≥6 months, are critical for preventing SARS-CoV-2 infection and subsequent illness and for reducing the public health impact of post-COVID symptoms and conditions.

**Summary**

**What is already known about this topic?**

Children and adolescents might be at risk for certain post-COVID symptoms and conditions.

**What is added by this report?**

Compared with patients aged 0–17 years without previous COVID-19, those with previous COVID-19 had higher rates of acute pulmonary embolism (adjusted hazard ratio = 2.01), myocarditis and cardiomyopathy (1.99), venous thromboembolic event (1.87), acute and unspecified renal failure (1.32), and type 1 diabetes (1.23), all of which were rare or uncommon in this study population.

**What are the implications for public health practice?**

COVID-19 prevention strategies, including vaccination for all eligible persons aged ≥6 months, are critical to preventing SARS-CoV-2 infection and subsequent illness, and reducing the public health impact of post-COVID symptoms and conditions among persons aged 0–17 years.

## Footnotes

* CDC defines post-COVID conditions as new, returning, or ongoing health problems occurring ≥4 weeks after being infected with SARS-CoV-2. https://www.cdc.gov/coronavirus/2019-ncov/long-term-effects/index.html

† This analysis used CDC-licensed HealthVerity, Inc. medical claims data linked to SARS-CoV-2 commercial laboratory data (May 2022 release). Patients were eligible for inclusion in CDC licensed data if they had a health care encounter (diagnosis, procedure, treatment, or laboratory test) possibly related to COVID-19 on or after December 1, 2019.

§ A retrospective cohort of children and adolescents aged 0–17 years with continuous enrollment in an insurance plan during March 1, 2019–January 31, 2022, was identified within a subset of CDC-licensed HealthVerity data that included persons with a health care encounter possibly related to COVID-19. Patients with COVID-19 were selected from among patients with a positive SARS-CoV-2 test result during March 2020–November 2021 or an ICD-10-CM code of B97.29 (other coronavirus as the cause of diseases classified elsewhere) during March–April 2020 or U07.1 code (COVID-19, virus-identified [laboratory-confirmed]) during April 2020–November 2021 (https://www.cdc.gov/nchs/data/icd/Announcement-New-ICD-code-for-coronavirus-3-18-2020.pdf). Patients without COVID-19 were selected after excluding patients who had any ICD-10-CM codes related to COVID-19 (A41.89, B34.2, B97.21, B94.8, J12.81, J12.82, J12.89, M30.3, M35.81, U07.1, or U07.2), a positive SARS-CoV-2 test result, or received treatment for COVID-19 (casirivimab/imdevimab, etesevimab/bamlanivimab, sotrovimab, bebtelovimab, nirmatrelvir, molnupiravir, or remdesivir) at any point during the study period. Vaccination status of patients was not included for this analysis.

¶ The index date for the group of patients with COVID-19 was the date of either the first claim with a COVID-19 diagnosis code or the first positive SARS-CoV-2 test result (whichever occurred first). The index date for patients without COVID-19 in the main analysis was the date of a randomly selected claim during the month in which the patient without COVID-19 was matched to a patient with COVID-19. The index date for patients without COVID-19 in the sensitivity analysis was the date of the first negative SARS-CoV-2 test result, first health care encounter possibly related to COVID-19 (associated with an ICD-10-CM code of B97.89, Z86.16, R05, R06.02, R50.9, R19.7, R53.8, R09.3, R04.2, R09.2, J00–J06, J09–J11, J12.9, J13–J18, or J80), or the first claim during the pandemic period if other dates were not available.

** Potential post-COVID conditions were selected from a range of body systems and were assessed by the first occurrence of at least one of the following ICD-10-CM codes documented 31–365 days after the index date but not during the 7–365 days preceding the index date: 1) circulatory system disorders: acute pulmonary embolism (I26), myocarditis and cardiomyopathy (A36.81, B33.20, B33.22, B33.24, B58.81, I25.5, I40, I41, I42.0–I42.5, I42.8, I42.9, I43, I51.4, J10.82, J11.82, and O90.3), cerebrovascular disease (G46 and I67–I68 [except I67.0 and I67.4]), venous thromboembolic event (I82.40, I82.49, I82.4Y, I82.4Z, I82.62, I82.50, I82.59, I82.5Y, I82.5Z, and I82.72), cardiac dysrhythmias (I47, I48.0, I48.19, I48.21, I48.3–I48.9, and I49.1–I49.9); 2) endocrine, nutritional, and metabolic disorders: type 1 diabetes (E10), type 2 diabetes (E11); 3) digestive system disorders: gastrointestinal and esophageal disorders (K20, K21, K22.0–K22.6, K22.89, K22.9, K23, K58, K59.0–K59.2, K59.89, K59.9, and K92.9); 4) musculoskeletal and connective tissue disorders: muscle disorders (M60.0, M60.1, M60.8, M60.9. M61, M62, and M63); 5) mental, behavioral, and neurodevelopmental disorders: anxiety and fear-related disorders (F06.4, F40.0, F40.1, F40.228, F40.230, F40.231, F40.232, F40.233, F40.240, F40.248, F40.8, F40.9, F41, and F93.0), mood disorders (F06.30, F34.8. F34.9, and F39); 6) nervous system disorders: neurological conditions (F05, R40.0, R41, R44, A85, A86, G04, G05, R29, R26, R27, G26, and G50–G65); 7) respiratory system disorders: asthma (J45); 8) genitourinary disorders: acute renal failure (N17 and N19), chronic kidney disease (N18 and R88.0); 9) blood system disorders: coagulation and hemorrhagic disorders (D47.3, D65, D68.3–D68.9, D69, D75.82, D75.83, and M36.2); 10) other symptoms, signs and abnormal clinical and laboratory findings: malaise and fatigue (G93.3, R53.1, and R53.8), respiratory signs and symptoms (R04–R09), smell and taste disturbances (R43.8 and R43.9), symptoms of mental conditions (F30.4, F31.70, F31.72, F31.74, F31.76, F31.78, F32.5, F33.40, F33.42, R45.0–R45.7, R45.8 [except R45.851 and R45.88], and R46), sleeping disorders (G47 and R06.3), circulatory signs and symptoms (R00, R01, R03.0, and R09.89), dizziness and syncope (I95.1, G90.9, R42, and R55), and musculoskeletal pain (M25.5, M25.6, M54.6, M54.8, M54.9, M79.1, M79.6, and M79.7).

†† Proportional hazards assumption was tested for every Cox proportional hazards model. In some models, proportionality assumption for certain variables was rejected, and the Schoenfeld residuals and survival curves were visually examined. Although slopes were not always parallel, the survival curves did not cross in any cases, indicating that the nonproportionality might not lead to severe bias in the results. The estimated aHRs from Cox models were compared against aHRs from Weibull models (estimated using “SurvRegCensCov” R package) and average aHRs from weighted Cox models (estimated using “coxphw” R package) and were found to be very close in magnitude and significance level.

§§ Previous hospitalization was defined by a presence of an inpatient claim during the 7–365 days before the index date. Previous medical complexity was defined using the validated pediatric medical complexity algorithm as presence of complex chronic disease (at least one claim with a progressive condition, at least one claim with malignant neoplasms, or at least one claim per body system for two different body systems), presence of noncomplex chronic disease (at least one claim for a single body system not flagged as progressive), or absence of chronic disease (reference category; none of the previously described encounters) during the 7–365 days before the index date.

¶¶ Age-stratified analyses were only performed when there were at least 10 patients with COVID-19 and at least 10 patients without COVID-19 in that age group with the specific symptom or condition. Each model was adjusted for age as a continuous covariate (to account for age differences within each age group), sex, race, U.S. Census Bureau region, payor type, previous medical complexity, and previous hospitalization.

*** The maximum possible matching ratio was used. For the main analysis, patients without COVID-19 were matched 3:1 to patients with COVID-19. For the analysis of a cohort of patients with no previous symptoms or conditions of interest, patients without COVID-19 were matched 2:1 to patients with COVID-19.

††† 45 C.F.R. part 46.102(l)(2), 21 C.F.R. part 56; 42 U.S.C. Sect. 241(d); 5 U.S.C. Sect. 552a; 44 U.S.C. Sect. 3501 et seq.
